# An examination of an adapted Project ECHO model series during the COVID‐19 pandemic in Idaho

**DOI:** 10.1002/puh2.128

**Published:** 2023-09-30

**Authors:** Jonathan D. Moore, Julio de Leon Gonzalez, Madeline P. Casanova, Kathleen Rodgers, Russell T. Baker

**Affiliations:** ^1^ WWAMI Medical Education Program University of Idaho Moscow Idaho USA; ^2^ Idaho Office of Rural and Underserved Medical Research University of Idaho Moscow Idaho USA; ^3^ Project ECHO University of Idaho Boise Idaho USA

**Keywords:** COVID‐19, evaluation, Project ECHO, rural medicine, telementoring

## Abstract

**Introduction:**

Idaho is a medically underserved and rural state comprised mostly health professional shortage areas for primary and specialty care. Given the state shortages and barriers to access care, the testing and treatment of COVID‐19 may be improved through the implementation of telementoring education programs such as Project Extension for Community Healthcare Outcomes (ECHO). The purpose of this article was to assess session attendance and self‐reported healthcare clinician perceptions of the ECHO Idaho COVID‐19 series (2020 and 2021), a modified Project ECHO model provided during the COVID‐19 pandemic in Idaho.

**Methods:**

ECHO Idaho developed a COVID‐19 ECHO series for healthcare providers to attend in 2020 and 2021. The sessions included COVID‐19 updates specific to Idaho, didactic presentations on prevention, screening, diagnosis, and evidence‐based treatments for COVID‐19, and case‐based presentations.

**Results:**

A total of 664 individuals attended the 2020 series and 260 individuals attended the 2021 series, with 1752.5 continuing medical education hours being claimed. Participants reported series participation increased overall knowledge of COVID‐19, knowledge of best practices for treatment, and awareness of resources available in Idaho. Further, series participation was perceived to reduce feelings of isolation, enhance access to information, and support healthcare professionals (e.g., provided resources) during the pandemic.

**Conclusion:**

Results of this evaluation indicate that the ECHO Idaho COVID‐19 series was a useful and valuable program to implement during a pandemic in a rural and frontier state to improve physician knowledge and specialty training.

## Graphical abstract

**Graphical Abstract**: *Research objective*: Idaho is a medically underserved and rural state, comprised primarily health professional shortage areas. Telehealth programs, such as Project ECHO Idaho, are necessary to bolster provider knowledge and improve access to care. The objective of this study was to evaluate self‐reported perceptions of healthcare professionals who participated in the 2020 and 2021 ECHO Idaho COVID‐19 series. *Methods*: ECHO Idaho developed a COVID‐19 series for healthcare providers to attend in 2020 and 2021. The sessions included COVID‐19 updates specific to Idaho, didactic presentations on prevention, screening, diagnosis, and evidence‐based treatments for COVID‐19, and case‐based presentations. *Key findings*: Participating healthcare professionals reported an increase in overall knowledge of COVID‐19, knowledge of best practices for treatment, and awareness of resources available in the state of Idaho. Results of this evaluation indicate that the ECHO Idaho COVID‐19 series was a useful and valuable program to implement during a pandemic in a rural and frontier state to improve provider knowledge and specialty medical training.

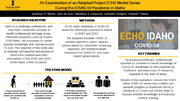

## INTRODUCTION

The World Health Organization declared the coronavirus outbreak a public health emergency of international concern on January 30, 2020 [1], and the onset of the SARS‐CoV‐2 (COVID‐19) pandemic was an unprecedented challenge to the United States (US) healthcare system [2]. The first case of COVID‐19 in Idaho was reported on March 13, 2020 [3]. Soon after, the incidence of cases in Idaho increased to over 100 new cases per day by early April 2020 [4], further straining the state's healthcare system. Idaho is a medically underserved state with low physicians per capita, with provider shortages being compounded by a rapidly growing population [5, 6].

Idaho is considered medically underserved having 98.7% health professional shortage areas for primary care [5]. Additionally, the majority of Idaho is considered rural or frontier; 35 out of 44 counties are classified as such [7]. The limited numbers of providers for Idaho residents and challenges (e.g., geographical isolation) to accessing care in a predominately rural and frontier state create challenges for healthcare providers to provide proper treatment to patients [5, 7]. These challenges were exacerbated by the COVID‐19 pandemic, which escalated provider burnout and overwork [8]. Additionally, rural physicians across Idaho lacked access to readily available specialists and had to care for patients using limited resources and information. The need to stay up to date with best practices while being overwhelmed with patients and cases in their community created obstacles for care. Such circumstances exposed a need to develop a sustainable, regularly occurring program to reach providers across the state to provide timely information while reducing provider barriers to attendance [9].

The Project Extension for Community Healthcare Outcomes (ECHO) model, developed by the University of New Mexico Health Sciences Center, uses telehealth technology, case‐based learning, interdisciplinary team‐based development, and best practice protocols to assist healthcare providers in developing knowledge [10]. ECHO Idaho, established in 2018, is the only Project ECHO replication partner based in Idaho, providing healthcare providers in rural and underserved Idaho communities with educational opportunities to engage in specialty training [10]. Thus, ECHO Idaho was well‐positioned to develop and deliver a just‐in‐time COVID‐19 telementoring session in March 2020 to provide rural physicians with the most current information on how to care for patients with COVID‐19 [9]. This rapidly deployed, adapted, and modified ECHO session was perceived by participants as a useful tool to relay information to promote best practices at the start of the pandemic and provided clinicians with a support system to connect and discuss the effects of the pandemic [9].

Participant responses to the just‐in‐time session identified a need for continued support and further training for healthcare providers during the COVID‐19 pandemic. Thus, ECHO Idaho developed and provided ongoing COVID‐19 sessions from March 24, 2020, to August 24, 2021, using an adapted ECHO model within a previously established Project ECHO replication partner network. The ECHO Idaho sessions included Idaho‐specific COVID‐19 updates (e.g., epidemiology), as well as tele‐education (i.e., didactic presentations about prevention, screening, diagnosis, and evidence‐based treatments and case‐based learning) [11] and tele‐mentoring (e.g., mentorship and guidance from an interdisciplinary expert Idaho‐based panel) adapted using the Project ECHO model [11]. Although the Project ECHO model has been reported to be effective at increasing knowledge and competence for specialty conditions [12, 13, 14], more research is needed to assess the ability to use a previously established Project ECHO network to deliver an adapted ECHO series that can effectively provide support throughout a pandemic in a rural state. Therefore, the purpose of this article was to assess session attendance and self‐reported healthcare clinician perceptions of the ECHO Idaho COVID‐19 series (2020 and 2021), a modified Project ECHO model provided during the COVID‐19 pandemic in Idaho.

## METHODS

### Study setting

The Project ECHO Idaho program was conducted through the University of Idaho WWAMI (Washington, Wyoming, Alaska, Montana, Idaho) Medical Education Program as a replication partner of the Project ECHO program initiated at the University of New Mexico. ECHO Idaho was created in March of 2018 for the purpose of expanding healthcare provider education and ultimately improving access to care for residents of the rural state of Idaho, US [9, 11, 12].

### Series description

The ECHO Idaho COVID‐19 series was a tele‐education/telementoring health education series that used didactic presentations and case‐based presentations to educate Idaho healthcare providers on the COVID‐19 pandemic topics related to prevention, screening, diagnosis, and evidence‐based treatments.

The series took place between March 2020 and August 2021 using Zoom software (Zoom Video Communications, Inc.). A total of 58 1‐h sessions occurred; 32 sessions were held for the 2020 COVID‐19 ECHO series, and 26 sessions were held for the 2021 series. The first 30 min of a session included an educational lecture, and the last 30 min were reserved for case‐focused discussions and participant question‐and‐answer. The case‐focused discussions were presented by an interdisciplinary Idaho‐based clinical panel, and recommendations for care were discussed by both the session participants and the clinical panel. The educational lectures and presenters were predominantly chosen by the clinical specialists, with various topics (e.g., outpatient/emergency department care, inpatient/critical care, underserved communities in a pandemic, and pediatric considerations) being presented to meet the needs of the Idaho healthcare community. The series was provided to attendees at no cost, and continuing medical education (CME) credit was offered to all attendees; attendees could claim CME credit and a certificate of completion for each session attended.

### Respondent recruitment

The ECHO Idaho COVID‐19 series was intended for Idaho healthcare clinicians (e.g., physicians, physician assistants [PAs], nurse practitioners, and nurses) who were providing care to COVID‐19 patients. Participation was not limited, however, and participant recruitment included other members of the healthcare team (e.g., administrators and public health professionals). ECHO Idaho COVID‐19 sessions were advertised across the ECHO Idaho network (e.g., prior attendee contact lists and program website), Idaho state healthcare association networks and organizations, and residency and health profession training programs in Idaho.

### Post‐session evaluation survey

A mixed‐methods survey was developed in Qualtrics (Qualtrics, LLC) using both qualitative and quantitative research questions. The survey was designed by members of the Idaho Office of Underserved and Rural Medical Research to meet CME accreditation requirements and provide an assessment of the ECHO Idaho COVID‐19 series. Survey items were developed after reviewing prior ECHO Idaho surveys, preexisting survey tools from other ECHO programs, and COVID‐19 evaluation program literature. A five‐member panel reviewed the items, and the survey was altered to improve content, item design, and clarity until a final version of the instrument was approved. The post‐session survey included demographic (e.g., profession, credentials, and primary practice location), CME (e.g., credits claimed), quantitative (e.g., Likert scale items), and qualitative (i.e., four open‐ended questions) questions. A 5‐point Likert scale (strongly disagree/extremely dissatisfied = 1, strongly agree/extremely satisfied = 5) was used that included a “not applicable” response option when appropriate (e.g., a session attendee was not a healthcare provider). Mean responses were determined to be high if they exceeded a value of 4. Survey items were designed to evaluate respondent perceptions of session benefits (e.g., knowledge change, peer network development, and improved patient care), session satisfaction, and areas for improvement using a mixed‐methods approach.

### Procedures

Attendance for the COVID‐19 series was recorded by ECHO Idaho staff and entered into iECHO, an online program management database [15]. After the conclusion of each ECHO Idaho COVID‐19 session, the link to a post‐session evaluation survey was provided to participants in the Zoom chat; the survey link was also available on the ECHO Idaho website and was included in ECHO Idaho emails about the COVID‐19 sessions. Survey completion was monitored by a member of the ECHO Idaho team to allow for the processing of CME certificates and programmatic quality improvement opportunities.

### Data analysis

The Qualtrics survey data were downloaded and exported for analysis to Statistical Package for Social Sciences (SPSS, V. 25.0, IBM). Frequency analyses were conducted on demographic data, whereas descriptive statistics (mean, standard deviation) were calculated for all Likert scale items. Only cases with fewer than 10% missing responses to learning outcome questions (i.e., Likert‐scale items) were retained for analysis; incomplete cases were removed for analysis. The qualitative analysis was conducted using a general inductive approach [16].

### Ethical considerations

All participants provided informed consent prior to study participation. The project was certified exempt by the University Institutional Review Board. Our survey data were stored on secured servers, only accessible by members of our research team, and data were used only for the purposes of this research study.

## RESULTS

### COVID‐19 series attendee demographics

A total of 924 unique participants (2020 participants = 664; 2021 participants = 260) attended at least 1 of the 58 total sessions held across the 2 years (2020 sessions = 32; 2021 sessions = 26); the average number of 2020 participants per session was 85 (range = 47–185 participants), whereas the average number of 2021 participants per session was 60 (range = 34–123 participants; Appendix I). A total of 1752.5 CME hours were claimed across the 58 sessions (2020 participants = 928.5 h; 2021 participants = 824 h). Most participants were from Idaho (2020 = 92.8%; 2021 = 93.1%), and all Idaho public health districts were represented. The 2020 sessions included participation from 67 different cities and 282 different health organizations, whereas the 2021 session included participants from 34 cities and 127 healthcare organizations. Physicians (2020 = 25.2%; 2021 = 16.5%) were the largest health profession who participated; a full breakdown of participant demographics is presented in Table 1.

**TABLE 1:** Demographic characteristics of respondents from the 2020 to 2021 Extension for Community Healthcare Outcomes (ECHO) COVID‐19 series in Idaho, USA.

	2020 attendees *n* (%)	2021 attendees *n* (%)	2020 survey respondents *n* (%)	2021 survey respondents *n* (%)
**Profession**				
Physician	167 (25.2)	43 (16.5)	400 (43.10)	282 (45.05)
Physician assistant	41 (6.2)	7 (2.7)	93 (10.02)	27 (4.31)
Nurse practitioner	34 (5.1)	14 (5.4)	30 (3.23)	16 (2.56)
Pharmacist	21 (3.2)	4 (1.5)	22 (2.37)	2 (0.32)
Nurse	58 (8.7)	52 (20.0)	138 (14.87)	145 (23.16)
Social worker	57 (8.6)	24 (9.2)	127 (13.69)	61 (9.74)
Counselor	23 (3.5)	5 (1.9)	44 (4.74)	21 (3.35)
Other	263 (39.6)	111 (42.7)	74 (7.97)	72 (11.50)
**Idaho public health district (PHD)**				
PHD 1	49 (8.0)	36 (14.8)	91 (11.26)	41 (7.85)
PHD 2	87 (14.1)	22 (9.0)	74 (9.16)	40 (7.66)
PHD 3	45 (7.3)	12 (4.9)	62 (7.67)	46 (8.81)
PHD 4	304 (49.4)	128 (52.5)	352 (43.56)	252 (48.28)
PHD 5	40 (6.5)	16 (6.)	34 (4.21)	38 (7.28)
PHD 6	58 (9.4)	21 (8.6)	83 (10.27)	37 (7.09)
PHD 7	32 (5.2)	9 (3.7)	112 (13.86)	68 (13.03)
**Ethnicity**				
Caucasian/White			908 (94.88)	581 (91.50)
African American/Black			4 (0.42)	16 (2.52)
Hispanic/Latino			11 (1.15)	11 (1.73)
Asian/Pacific slander			18 (1.88)	13 (2.05)
Other			16 (1.67)	14 (2.20)
**Sex**				
Men			413 (43.75)	291 (46.63)
Women			519 (54.98)	318 (50.96)
Other			2 (0.21)	0 (0)
Prefer not to answer			10 (1.06)	15 (2.40)

### Post‐session evaluation survey respondents

Demographic characteristics for the 2020 and 2021 ECHO Idaho COVID‐19 post‐session evaluation respondents are presented in Table 1 with total sample sizes of 928 and 626, respectively. For the 2020 analysis, the highest percentage of respondents (43.10%) were physicians who practiced in Idaho's fourth public health district (43.56%), and reported being Caucasian/White (94.88%) and female (54.98%). Similarly, for the 2021 series, the highest percentages of respondents were physicians (45.05%) who practiced in Idaho's fourth public health district (48.28%), and reported being Caucasian/White (91.50%) and female (50.96%).

Results for the 2020 and 2021 ECHO Idaho COVID‐19 series are presented in Table 2. Respondents reported the highest level of agreement with the item “The ECHO COVID‐19 session … supported my work as a healthcare professional” (4.46 and 4.47, respectively; high level of agreement >4.00). However, respondents reported a high level of agreement for all items (>4.00), with mean values ranging from 4.20 to 4.46 for 2020 and 4.23 to 4.47 for 2021. Table 3 presents results related to respondents’ level of improved preparedness due to participation in the ECHO Idaho COVID‐19 series. For the 2020 data, the highest level of improvement in preparedness was reported for the item “I am better prepared to … meet the healthcare needs of my community” (4.47). For the 2021 data, the highest levels of improvement in preparedness were reported for the items “I am better prepared to … improve my client/patient care” (4.47) and “I am better prepared to … provide recommended care to clients/patients” (4.47). However, respondents reported a high level of agreement for all items (>4.00) with values ranging from 4.43 to 4.47 for 2020 and 4.42 to 4.47 for 2021. In addition, respondents reported overall satisfaction with the ECHO Idaho COVID‐19 series for both the 2020 (4.82) and 2021 series (4.87) with a mean value >4 indicating satisfaction.

**TABLE 2:** Participant agreement to statements about their participation in the 2020 and 2021 Extension for Community Healthcare Outcomes (ECHO) COVID‐19 series in Idaho, USA (1 = strongly disagree, 5 = strongly agree).

	Year of series	
The ECHO COVID‐19 session …	2020	2021
	Mean ± SD	Mean ± SD
Increased my overall knowledge of COVID‐19	4.44 ± 0.74	4.53 ± 0.68
Improved my understanding of referral information for patients/individuals	4.20 ± 0.86	4.23 ± 0.83
Enhanced my knowledge of best practices regarding treatment options or individuals/patients	4.39 ± 0.78	4.46 ± 0.74
Made me more aware of resources available to support patients/individuals	4.35 ± 0.83	4.32 ± 0.81
Reduced my feelings of professional isolation in dealing with the COVID‐19 pandemic	4.31 ± 0.85	4.24 ± 0.87
Provided a valuable peer network for me to utilize moving forward in my clinical practice	4.30 ± 0.84	4.28 ± 0.82
Presented immediate access to valuable information that enhances patient care in my state	4.43 ± 0.78	4.43 ± 0.72
Supported my work as a healthcare professional	4.46 ± 0.78	4.47 ± 0.69

**TABLE 3:** Descriptive statistics of improved perceptions of preparedness due to participation in the 2020 and 2021 Extension for Community Healthcare Outcomes (ECHO) COVID‐19 series in Idaho, USA (1 = strongly disagree, 5 = strongly agree).

As a result of my participation in this ECHO COVID‐19 session, I am better prepared to …	2020	2021
Provide recommended care to clients/patients	4.44 ± 0.92	4.47 ± 0.82
Improve my client/patient care	4.44 ± 0.90	4.47 ± 0.82
Meet the healthcare needs of my clients/patients	4.44 ± 0.88	4.44 ± 0.83
Meet the healthcare needs of my community	4.47 ± 0.86	4.45 ± 0.80
Meet the healthcare of my state	4.43 ± 0.87	4.42 ± 0.83

### Benefits of participating in the series: qualitative assessment

A total of 888 survey responses (2020 responses = 568; 2021 responses = 320) were provided to the open‐ended question related to providers’ perceived clinical benefit of participating in the series. Six themes (Table 4) were observed for both 2020 and 2021 responses: (1) increased knowledge (2020 = 46.5% of responses; 2021 = 45.9% of responses), (2) provided beneficial information (2020 = 41.4% of responses’ 2021 = 43.1% of responses), (3) perceived future improvement in medical practice or teaching (2020 = 4.4% of responses; 2021 = 2.5% of responses), (4) increased awareness (2020 = 4.2% of responses; 2021 = 3.4% of responses), (5) provided helpful resources (2020 = 3.2% of responses; 2021 = 2.8% of responses), and (6) professional interconnectedness (2020 = 0.4% of responses; 2021 = 2.2% of responses). Examples of participants’ responses are provided in Table 4.

**TABLE 4:** Qualitative analysis of respondents’ reported benefits of participating in the 2020 and 2021 COVID‐19 Extension for Community Healthcare Outcomes (ECHO) Series in Idaho, USA.

Year	Main themes	Example of response
2020	Professional interconnectedness	“Feeling a part of a community of caring healthcare providers”
	Provided beneficial information	“Excellent review of healthcare worker burnout and stress secondary to COVID pandemic”
	Increased awareness	“Increased my awareness of medications and treatment”
	Increased knowledge	“Increase my knowledge on medications used to treat COVID‐19″
	Perceived future improvement in medical practice or teaching	“Improve self care and therefore better care for those I treat”
	Provided helpful resources	“Once again I am uplifted with the resources that this session has given me as well as my holistic approach to helping pts. and students”
2021	Professional interconnectedness	“Feeling a connection to others that are struggling with similar situations. Reinforcing the continued need for prevention”
	Provided beneficial information	“Good information for staff and clients”
	Increased awareness	“increased my awareness on covid cases being treated in hospitals”
	Increased knowledge	“increased knowledge of COVID vaccines”
	Perceived future improvement in medical practice or teaching	“better able to serve the needs of clients wanting information about COVID concerns to support them talking to their medical providers for specific concerns re: their age and recent state announcements”
	Provided helpful resources	“Reminders of a full assessment of resources and linking to resources available‐”

## DISCUSSION

ECHO Idaho develops training programs to address healthcare providers’ clinical practice knowledge and competence shortcomings related to specialty conditions in Idaho, with a focus on meeting the needs of Idaho clinicians working in rural and frontier communities [13, 17]. The COVID‐19 pandemic brought rapidly changing recommendations for best practices regarding personal protective equipment, vaccinations, and treatments [18], prompting ECHO Idaho to launch an adapted Project ECHO model for healthcare providers [9]. The purpose of this article was to conduct an assessment of the program by assessing session attendance and self‐reported healthcare clinician perceptions of the ECHO Idaho COVID‐19 series to provide education and training during a pandemic.

The ECHO Idaho COVID‐19 series was far reaching with higher total unique attendance (926 participants) than prior ECHO Idaho series for behavioral health (178 participants) [12] or opioid and addiction treatment (273 participants) series [13]. Per session attendance was also high, with averages of 85 (range = 47–185) participants per session in 2020 and 60 (range = 34–123) participants per session in 2021. The per session attendance was substantially higher than prior reports on other regularly occurring ECHO series in Idaho: A behavioral health series had an average of 26 attendees (range = 12–44) per session, whereas an opioid use disorder reported 22.8 attendees (range = 11–44) per session [12, 13]. The averages of the COVID series were also higher than those of a multiyear short‐duration (i.e., 10‐week series) perinatal substance use disorder series ECHO Idaho program that reported 44.3 and 31.6 attendees per session for the 2020 and 2021, respectively [17]. Like the prior ECHO Idaho series [12, 13, 17], the COVID‐19 series had participation from individuals from all seven public health districts; thus, providers residing in more rural and frontier areas of Idaho were also able to participate throughout the pandemic.

The high attendance, however, was expected as prior research indicated that future COVID‐19 programming was needed and would be well attended by Idaho healthcare providers [9]. Despite the increase in total participation and the need through the region, the majority (93%) of attendees continued to reside in Idaho, which was similar to the Idaho resident demographic makeup of previous ECHO Idaho series [13, 17]. The high attendance rate may have been influenced by the far‐reaching effect of COVID [19]. Specifically, this series saw attendance increases (total number and percentage) among key health professions (i.e., physicians, nurses, and PAs) responsible for providing care during the pandemic (Table 1) compared to prior ECHO Idaho series [9, 12, 13, 17]. Additionally, COVID‐19 ECHO programming through the University of New Mexico (June 1, 2020 through May 31, 2021) also had high total unique participation (1421 attendees) [20], whereas a weekly series in Oklahoma had over 500 unique participants and averaged 20 participants per session [20]. The high attendance across the various Project ECHO COVID‐19 programs highlights the need for such programming during the pandemic [20]; the attendance data and demographic profiles of those attending ECHO Idaho programming indicate that the various series were able to target participation from the relevant health professions populations depending on the series topic [9, 12, 13, 17].

Overall, respondents expressed satisfaction with the training sessions, and most agreed that the ECHO COVID‐19 sessions increased their overall knowledge of COVID‐19, enhanced their knowledge of best practices regarding treatment options, made them aware of resources available in Idaho, and provided immediate access to valuable information to enhance patient care in their community. Previous ECHO Idaho series have reported high satisfaction with their participation [12], as well as increased knowledge of specialty health conditions [13]. Similarly, a Connecticut COVID‐19 ECHO series reported 93%–94% of respondents stated they gained new knowledge due to participation in the session [14] which is similar to previous ECHO Idaho research which found that participants reported high agreement to the statement that the ECHO COVID‐19 session increased their overall knowledge of COVID‐19 [9]. Additionally, similar to the current study, most participants (96%–97%) from the Connecticut series reported satisfaction with the case‐based presentation and didactic components of the sessions [14]. Furthermore, in another COVID ECHO series conducted in New Mexico, participants reported feeling that their goals were reached from participation in the series [21]. Additionally, most respondents (68.2%) reported that they would definitely use what they learned in their practice and 22% reported that they would probably use what they learned for their practice [21].

In addition to providing valuable and immediate access to information about recommendations, resources, and practices during the pandemic, the ECHO Idaho COVID‐19 sessions also provided valuable support to the participants. Likert scale and qualitative responses supported the idea that attendance reduced professional isolation and provided beneficial peer networks, which is in alignment with previous literature that demonstrates the ECHO model reduces professional isolation [22, 23]. Respondents also reported feeling more prepared to provide better patient care and believed that sessions were able to help them meet the needs of their patients, community, and state throughout the pandemic. Similarly, previous ECHO series also observed session participants reporting feeling that their participation improved their clinical practice [12].

Although our study provides evidence that using an established Project ECHO network to develop an adapted ECHO program may be a beneficial tool to use during a pandemic to support healthcare professionals, our study does have some limitations. First, our study collected important self‐report data; however, we were unable to assess if a respondent's perceived increase in knowledge or competence to provide treatment led to changes in clinical practice or resulted in improved patient outcomes. Future research should focus on clinical practice, self‐efficacy, and competence changes of those who attend ECHO sessions. Second, although responses to the survey were similar to previous studies [12–14, 21], the information obtained may not be representative of all attendees’ experience in the ECHO Idaho COVID‐19 series. Future researchers should seek to obtain data from a larger proportion of individuals who attend sessions and may want to explore perceptions of nonresponders to assess nonresponse bias. It is possible that the survey response rate was influenced by ECHO Idaho programming occurring throughout a pandemic: Healthcare providers may have been unwilling to spend time away from patient care to complete a voluntary survey for CME credit; thus, research into the barriers and motivations to completing post‐session surveys during the COVID‐19 pandemic might be valuable. Finally, the ECHO Idaho programming did not use a cohort (i.e., limited session participation) or control group model due to the need to disseminate information to as many providers as possible during the pandemic. Future research studies using different approaches (e.g., waitlist‐control group) collecting different variables (e.g., patient outcomes) are needed to fully understand the effects of the ECHO model and program participation.

## CONCLUSION

The novel adaptation of the ECHO model to provide a COVID‐19 ECHO series in Idaho allowed valuable training, mentoring, and peer networking to be provided to Idaho healthcare professionals. Survey respondents reported participation prepared them to meet the needs of their patients and communities at a time when resources, information, and access were not otherwise readily available. Our findings support the use of the adapted Project ECHO model to develop and deliver specialized training to providers during a pandemic for the delivery of critical and new information to benefit healthcare providers.

## AUTHOR CONTRIBUTIONS

Jonathan D. Moore, Madeline P. Casanova, Kathleen Rodgers, Julio de Leon Gonzalez, and Russell T. Baker conceptualized the study. Jonathan D. Moore, Madeline P. Casanova, and Russell T. Baker performed data analysis and interpretation. Jonathan D. Moore, Julio de Leon Gonzalez, Madeline P. Casanova, and Russell T. Baker wrote article drafts. All authors were responsible for reviewing and editing the article. All authors approved the final version of the article.

## CONFLICT OF INTEREST STATEMENT

The authors declare that they have no conflicts of interest.

## ETHICS STATEMENT

The University of Idaho Institutional Review Board reviewed this project and certified it exempt (20‐086).

## Supporting information


Supporting Information

## Data Availability

The data that support the findings of this study are available from the corresponding author upon reasonable request.
