# Supplementary material for: An examination of an adapted Project ECHO model series during the COVID‐19 pandemic in Idaho
Source: Public Health Chall. 2023 Sep 30;2(4):e128. doi: 10.1002/puh2.128 (PMC12039751; doi:10.1002/puh2.128)
Supplement: Supplementary file 1 — Supporting Information [file PUH2-2-e128-s001.docx]

**Appendix I**

Table 5. Series Attendance during the 2020 and 2021 ECHO Idaho COVID-19 Series by session.

| **Year** | **Session** | **Title of Session** | **Attendance** |
| --- | --- | --- | --- |
|  |  |  |  |
| 2020 | Session 1 | Primary Care, Pre-Hospital, and Emergent Presentations of COVID-19 | 181 |
|  | Session 2 | COVID-19 in Inpatient Settings: Acute and Intensive Care | 106 |
|  | Session 3 | Microbiology of COVID-19 and the Infectious Disease Clinical Perspective | 185 |
|  | Session 4 | Goals of Care: Difficult Conversations and Clinical Palliative Care for COVID-19 Positive Patients | 93 |
|  | Session 5 | COVID-19 Case Conversation: Inpatient and Critical Care | 132 |
|  | Session 6 | COVID-19 Case Conversation: Outpatient/ED | 96 |
|  | Session 7 | COVID-19 Case Conversation: Inpatient and Critical Care | 102 |
|  | Session 8 | COVID-19 Case Conversation: Outpatient/ED | 88 |
|  | Session 9 | COVID-19: Pediatric Considerations | 94 |
|  | Session 10 | Behavioral Health in the Time of COVID-19 | 70 |
|  | Session 11 | COVID-19 Moving Forward, Moving Targets: Recovery and Rebound | 97 |
|  | Session 12 | COVID-19 and Health Equity: Underserved Communities in a Pandemic | 77 |
|  | Session 13 | Work and Play: Imagining an Idaho Summer | 88 |
|  | Session 14 | Balancing Parenting in the Unbalanced World of COVID-19 | 65 |
|  | Session 15 | Post-Acute Care and Residential Facilities: Challenges in the Time of COVID | 83 |
|  | Session 16 | Helping Others, Helping Ourselves: Lessons Learned in New Jersey | 58 |
|  | Session 17 | Operations Management for COVID-19 | 72 |
|  | Session 18 | COVID-19 Critical Care | 62 |
|  | Session 19 | COVID-19 Cases from the Infectious Disease Perspective | 83 |
|  | Session 20 | Palliative Care and COVID-19: A Framework for Discussion | 66 |
|  | Session 21 | COVID and School: Psychosocial Considerations | 72 |
|  | Session 22 | Travel-Related Transmission of COVID-19 | 81 |
|  | Session 23 | Self-Care for Healthcare During COVID-19 | 64 |
|  | Session 24 | Household Transmission of COVID-19 | 77 |
|  | Session 25 | Flu Vaccination in the Context of COVID-19 | 64 |
|  | Session 26 | Racism and Health: Widening Health Disparities in the COVID Era | 55 |
|  | Session 27 | Treatment Updates and Case Management | 66 |
|  | Session 28 | COVID-19 Vaccine Developments | 59 |
|  | Session 29 | Transmission, Testing, and Isolation: Exploring the Evidence | 81 |
|  | Session 30 | Medication Updates and Pearls for Outpatient Care | 73 |
|  | Session 31 | Laboratory Studies in COVID-19: Evaluating and Monitoring | 70 |
|  | Session 32 | Battling Burnout and Compassion Fatigue During COVID-19 | 47 |
| 2021 | Session 1 | Acute Complications of COVID-19 | 74 |
|  | Session 2 | COVID-19 Vaccine Rollout: Discussion and Q&A | 80 |
|  | Session 3 | COVID-19 Clinical Care in the Era of Vaccines and Variants | 86 |
|  | Session 4 | Treatment Updates for Ambulatory Care in Idaho | 47 |
|  | Session 5 | SARS CoV2 Round-Robin: From Variants to Long-Haulers and More | 55 |
|  | Session 6 | Vaccination Decisions: Strategies for Resolving Disagreements | 52 |
|  | Session 7 | Inpatient Telehealth Use in the Age of COVID | 39 |
|  | Session 8 | Long-COVID | 47 |
|  | Session 9 | Lab Studies in COVID-19: Evaluating and Monitoring | 43 |
|  | Session 10 | Motivational Interviewing for Behavior Change in a Post-COVID Era | 41 |
|  | Session 11 | COVID-19 Update June 2021 | 45 |
|  | Session 12 | MIS-C and Motivational Interviewing for Behavior Change in a Post-COVID Era | 34 |
|  | Session 13 | COVID-19 in Idaho: Summertime Steps to Prepare for Fall | 123 |
|  | Session 14 | Case Conversations: Delta in the Panhandle | 78 |
|  | Session 15 | The State We're In: Pandemic Ethics and Critical Care COVID | 57 |
|  | Session 16 | Delta Virulence and Antibody Treatment Updates | 69 |
|  | Session 17 | Review of Tx Literature | 46 |
|  | Session 18 | COVID Case by Case | 53 |
|  | Session 19 | The Impact of Vaccination and a case of COVID Encephalitis | 57 |
|  | Session 20 | Hospitals at Home: Strategies for Non-Hospital Acute Care | 52 |
|  | Session 21 | Palliative and Supportive Care during COVID-19 Surges | 50 |
|  | Session 22 | Post-Acute COVID-19 Syndrome: Pulmonary Complications | 62 |
|  | Session 23 | What to expect after the surge: Can an oral antiviral change the course of the pandemic? | 59 |
|  | Session 24 | ACIP Recommendations: COVID-19 and Beyond | 56 |
|  | Session 25 | Providing Care for the Unvaccinated: Psychological Approaches to Address Anger and Frustration | 76 |
|  | Session 26 | COVID-19: Looking Back, Looking Forward | 78 |
